# Shaping Minds, Changing Attitudes: A Systematic Review of the Impact of Different Teaching Interventions Regarding Attitudes and Knowledge Towards Electroconvulsive Therapy (ECT) Within Pre-Registration Healthcare Students

**DOI:** 10.1192/bjo.2025.10267

**Published:** 2025-06-20

**Authors:** Jasleen Deol, Naduni Jayasinghe, Anna Walters, Sanat Kulkarni

**Affiliations:** 1 Black Country Healthcare NHS Foundation Trust, Dudley, United Kingdom; 2 Aston University, Birmingham, United Kingdom; 3 Oxford Health NHS Foundation Trust, Oxford, United Kingdom; 4 Birmingham and Solihull Mental Health NHS Foundation Trust, Birmingham, United Kingdom; 5 Oxford University Hospitals NHS Foundation Trust, Oxford, United Kingdom

## Abstract

**Aims:** Electroconvulsive therapy (ECT) is an effective treatment modality used to manage a variety of different psychiatric conditions including treatment-resistant schizophrenia, depression and catatonia. Different teaching methods have been employed by educational institutions to teach healthcare students about ECT, however synthesis of this evidence is lacking. Several sources cite that there is negative stigma and attitudes towards ECT amongst Healthcare Professionals (HCPs). Teaching within undergraduate curricula may improve knowledge surrounding ECT, further reducing negative associations.

**Methods:** Using pre-determined search terms, a large language model was used to screen relevant databases (including ERIC and CINAHL), identifying 5,550 studies, 453 of which were duplications, leading to a total of 5,097 relevant studies. Pre-agreed strict inclusion and exclusion criteria were applied, and 19 studies were identified suitable for inclusion. Another 14 studies were reviewed again due to conflicting views, of which 7 were deemed suitable, totalling 26 relevant studies for inclusion. These texts were analysed in their entirety. Both qualitative and quantitative data was gathered, and this was heterogenous in nature. Qualitative data was thematically analysed.

**Results:** Diverse teaching techniques and interventions were identified, and these were successful to varying degrees. These interventions included: the development and creation of new educational modules centred around ECT; real time ECT demonstrations; teaching sessions paired with specialised technology enhanced learning interventions hosted remotely by consultant psychiatrists. A plethora of different interventions centred around improving knowledge of ECT amongst healthcare students varied in creativity, and even included a Hollywood depiction of ECT.

Educational interventions focusing on the improvement of students’ experience whilst on psychiatric placements correlated with a global positive improvement in knowledge levels regarding ECT. Video-based educational interventions were well received by students, and an improvement in attitude and knowledge regarding ECT was noted. Passive interventions, including didactic based teaching interventions described a notable positive shift in attitudes amongst students regarding ECT. However, some studies reported that the longevity of this improvement in knowledge and attitudes may be short-lived, affecting its translation to future medical practice.

**Conclusion:** This systematic review highlights the need to improve education of ECT amongst healthcare students, to ensure that future clinicians are well equipped with relevant knowledge concerning this important treatment modality. Ultimately interventions that strive to improve knowledge of ECT and induce positive experiences with students, helps to reshape attitudes towards this treatment modality and future clinical practice.

